# Intraocular pressure after phacoemulsification in patients with uncontrolled primary open angle glaucoma

**Published:** 2014-03-25

**Authors:** R Iancu, C Corbu

**Affiliations:** * Oftaclinic, Bucharest; ** Emergency Eye Hospital, Bucharest

**Keywords:** intraocular pressure, uncontrolled glaucoma, phacoemulsification

## Abstract

Abstract

Rationale (hypothesis). Although cataract and glaucoma represent an increasingly common situation encountered concomitantly, the management of this association is still debatable.

Objective (aim). We aimed to assess intraocular pressure dynamics after phacoemulsification in patients with uncontrolled primary open angle glaucoma (POAG).

Methods and Results. The present study was designed as a prospective, non-randomized, cohort study. The study population comprised of 38 patients with medically uncontrolled POAG who underwent cataract surgery by phacoemulsification between 2011 and 2012. Most of the patients (32/38, 84.2%) needed glaucoma surgery after a variable time (mean time between surgeries was 11.6 +/- 4.18 months). Mean preoperative IOP decreased with 2,1 +/- 3,7 mmHg at 6 months (CI 95% 1.96 to 3.56) and with 1,9 +/- 3,9 mmHg at 12 months compared with the baseline IOP. Postoperative IOP was statistically significant lower compared with its preoperative value at 6 months (p=9.11 x 10⁻⁸) and at one year (p=9.2 x 10⁻⁵). The difference between mean IOP at 6 months and 1 year after cataract surgery was not statistically significant (p>0.05). Preoperatively, all the patients received topical antiglaucoma therapy. After phacoemulsification, their number did not change statistically significant, but it showed a slight increase. Average number of topical glaucoma medications used preoperatively was 2.66 + / -0.66, while at 6 months after surgery it was 2.71 + / - 0,75 and at 12 months postoperatively, 2.9 +/- 0.53.

Discussion. IOP decreased statistically significant after phacoemulsification in patients with uncontrolled POAG, but the decrease was not sufficient for optimal glaucoma management; therefore, many patients needed subsequent glaucoma surgery.

## Introduction

Glaucoma and cataract are frequently encountered in the same patient, their prevalence increasing with age [**1**]. An increasing number of patients who present to the ophthalmologist with symptoms of cataract or glaucoma are diagnosed with both conditions [**2**]. Although it is an increasingly common situation, the management of combined cataract and glaucoma is still a subject of debate.

Primary open angle glaucoma (POAG) is one of the most common forms of glaucoma in adults. Nowadays, phacoemulsification represents the gold standard for cataract surgery. The surgical technique assumes small clear corneal incisions and foldable intraocular lenses, which greatly reduce the operating time [**3**]. Several studies report that cataract surgery lowers intraocular pressure (IOP) in normal and glaucomatous eyes [**4**-**6**] and some authors consider it a part of glaucoma management as well.

Recent studies suggest that lens extraction may be useful in eyes with primary angle closure glaucoma and may lead to significant IOP reduction [**7-**-**9**]. If for angle closure glaucoma, cataract surgery is considered a therapeutic option, for open angle glaucoma, things are not so clearly stated.

For patients with well-controlled POAG, with topical antiglaucoma medication, and symptomatic cataract, phacoemulsification with IOL, implantation is the best option. Cataract surgery rehabilitates vision and can also decrease IOP and the number of topical antiglaucoma medication.

Consequently, for patients with POAG and cataract, poorly controlled or uncontrolled with topical medication, there are several management options to be considered: staged approach (cataract surgery, followed or preceded by trabeculectomy) or combined approach (phacotrabeculectomy).

## Material and methods

Considering the high incidence and prevalence of cataract and glaucoma as separate clinical entities in general population, it seems reasonable to consider that the association of both conditions in the same patients is also common. Considering the great variety of visual function changes associated with glaucoma and cataract, there are well-established guidelines for each of them considered separately, but a consensus on the management of the patient with both conditions is lacking. Therefore, it is important to underline the effect of treating one of them on the concurrent disease progression. For clarity purposes, we selected only the patients with concomitant cataract and medically uncontrolled open angle glaucoma (POAG) (intraocular pressure over 21 mmHg with maximal medical therapy). The surgical treatment for the patients with cataract was the gold standard nowadays – phacoemulsification. 

In the present study, we aimed to assess the effect of phacoemulsification on intraocular pressure in patients with concomitant cataract and medically uncontrolled POAG. The primary endpoint was the change in intraocular pressure after phacoemulsification. Secondary endpoints were average time from the phacoemulsification to the glaucoma surgery, visual function after cataract surgery, complications rate, and the need for antiglaucomatous medication after cataract surgery. 

Study design 

The clinical study was designed as a prospective, non-randomized, cohort study. This study comprised of eyes with uncontrolled POAG and cataract surgically treated with phacoemulsification with IOL implantation, between 2011 and 2012. 

Patients’ selection 

 The inclusion criteria are listed in Table 1. Only one eye was considered for each patient. For patients with POAG affecting both eyes, the eye with higher IOP was selected, even if the cataract was more advanced in the other eye. 

All the patients presented the diagnostic criteria for POAG, IOP ≥ 21 mmHg, but ≤ 28 mmHg, with at least three topical glaucoma medication (POAG medically uncontrolled) and clinically significant lens opacities. Patients’ selection met the criteria in (**Table 1**). 

**Table 1:**
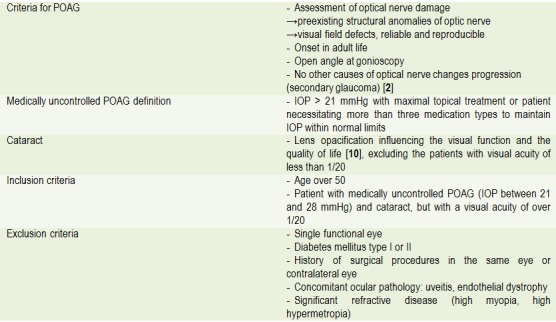
Inclusion and exclusion criteria

The study group consisted of patients with cataract and medically uncontrolled POAG who underwent phacoemulsification. All the patients enrolled met the inclusion criteria. Preoperatively, the patients were evaluated assessing the following parameters: IOP, target IOP, number and type of antiglaucomatous topical medication, corneal central thickness, the effect of topic treatment on ocular surface and its duration, Humphrey computerized perimetry indexes (MD, PD, VFI), visual acuity, depth of anterior chamber, gonioscopy, biomicroscopy of anterior and posterior pole, structural assessment of ocular nerve and nerve fibers layer.

 IOP evaluation

 The IOP was measured by using Goldmann tonometry, the gold standard for IOP assessment.

When quantifying the postoperative decrease in IOP, it is very important to clearly document the baseline IOP determined preoperatively. We considered as baseline IOP, the value estimated preoperatively with the following algorithm: the estimation was done by using at least two values measured in the last month before surgery and if the difference between measurements was less than 2 mmHg, the time interval between the valid measurements was of at least 5 days. If the difference between the two measurements was higher than 3 mmHg, we used the average value. This algorithm is similar with the one used in Ocular Hypertension Treatment Study [**11**,**12**].

Target IOP

After glaucoma was diagnosed, we calculated target IOP for each patient. Target IOP represents the value of IOP at which it is least likely that glaucoma will progress. Target IOP should be estimated according to glaucoma severity; for more severe glaucoma, we should consider smaller values for target IOP. Studies like Early Manifest Glaucoma Trial and Collaborative Initial Glaucoma Treatment Study conclude that target IOP should be with 25%-30% less than the baseline IOP [**13-**-**15**]. We calculated target IOP value extracting 30% from baseline IOP in each patient, but we also considered that this value should not be greater than 18 mmHg.

 Glaucoma medication (number and type)

To perform the statistical analysis, the number of topical antiglaucoma medication was numerically quantified, considering the number 1 for each topical pharmaceutical category. Thus, if patients used beta-blockers and topical prostaglandin analogs, it was coded with 2. There were also cases where patients used preoperatively systemic carbonic anhydrase inhibitors. Each tablet of acetazolamide 250 mg was coded with 2. 

Cataract surgery

The phacoemulsification surgery will be briefly described. All the cataract surgeries were performed through a small incision of 2.2 mm. The incisions were made in clear cornea, after corneal incision capsulorhexis being performed, followed by hydrodissection and hydrodelineation. The crystalline nucleus was split and removed by phacoemulsification. The irrigation-aspiration was used to remove any cortical fragments remaining after phacoemulsification. All the patients received a foldable intraocular lens, aspherical, monofocal or multifocal, placed inside the crystalline capsule (cases where intraocular implant was placed in sulcus were not included in the study). We practiced corneal wound closure in all the patients.

Postoperative follow-up

Postoperative reexaminations were performed after surgery with the following periodicity: one day, one week, one month, 3 months, 6 months, 9 months and 12 months after surgery. Postoperatively, the following parameters were assessed:

 - PIO

 - Number of topical antiglaucomatous medication

## Results

This study comprised 38 eyes with cataract and medically uncontrolled POAG. The mean age of the studied population was 71.7+/-8.27 years. The sex ratio was 61.5% females and 38.5% males.

 All the patients underwent cataract surgery (phacoemulsification with IOL implantation). The vast majority (32/38, 84.2%) also needed glaucoma surgery after a variable time (mean time between surgeries was 11.6 +/- 4.18 months). Only 6 patients out of 38 (15.8%) no longer required trabeculectomy after cataract surgery. The follow-up period considered in the study was the time between the cataract surgery and the glaucoma surgery in 32 patients, and for the rest of 6 patients, the mean follow up was 23 +/- 8,16 months. We evaluated all the patients at 6 months and one year; if the glaucoma surgery was performed, we considered as final IOP the pressure measured before trabeculectomy.

 Eyes were placed into 1 of 3 groups based on preoperative IOP as it follows: 28 to 27 mmHg (6 patients), 26 to 24 mmHg (13 patients), 23 to 21 mmHg (19 patients).

 Preoperative IOP varied between 21 and 28 mmHg. Mean preoperative IOP decreased with 2,1 +/- 3,7 mmHg at 6 months (CI 95% 1.96 to 3.56) and with 1,9 +/- 3,9 mmHg at 12 months compared with the baseline IOP.

**Fig. 1:**
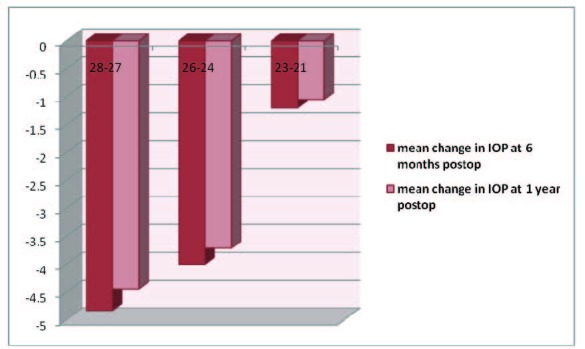
Mean IOP change at 6 months and 1-year follow-up, stratified on preoperative IOP values

 Postoperative IOP was statistically significant lower compared with its preoperative value at 6 months and at one year (**Table 2**). The difference between the mean IOP at 6 months and 1 year after cataract surgery was not statistically significant (**Fig. 1**).

**Table 2:**
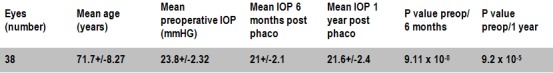
Mean IOP changes at 6 months and 1-year follow-up.

**Fig. 2:**
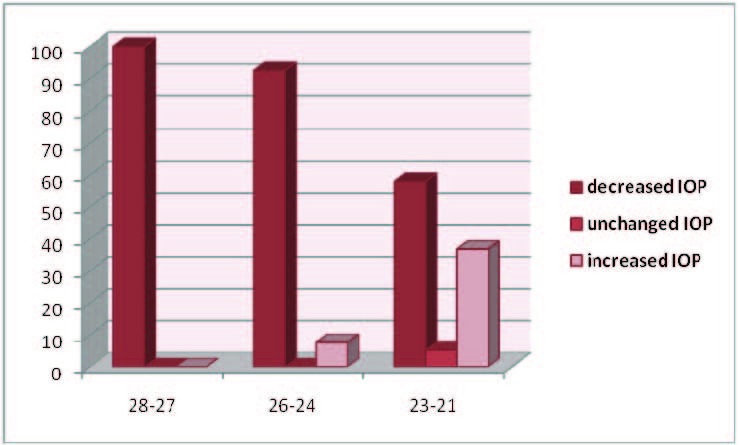
Distribution of postoperative IOP variation on preoperative IOP groups

Figure 2 depicts the distribution of IOP changes on preoperative groups of IOP. All the patients (100%) in the high preoperative IOP group showed a decrease in IOP. The group with the lowest preoperative IOP had the smallest number of patients with a decrease in IOP (58%) and the highest number of patients with increased IOP after surgery (37%).

Factors associated with postoperative intraocular pressure

Pearson correlation coefficient between postoperative IOP and demographic/clinical factors were as follows: mean preoperative IOP (r = 0.377, p <0.001), baseline age (r =0.07, p = 0.87), gender (r = 0.03, p = 0.23). The only factor significantly associated with postoperative IOP was preoperative IOP (p v0.001), although the correlation was weak (r=0.377). Higher preoperative IOP was associated with higher postoperative IOP while lower preoperative IOP was associated with lower postoperative IOP.

 Number of topical antiglaucoma treatment

 The average number of topical glaucoma medications used preoperatively was 2.66 + / -0.66, while at 6 months after surgery it was 2.71 + / - 0,75 and at 12 months postoperatively, 2.9 +/- 0.53. The difference between the values at 6 months and the ones at 12 months was not significantly different. Preoperatively, all the patients received topical antiglaucoma therapy. After phacoemulsification, their number did not change statistically significant, but it showed a slight increase.

Postoperatively, there was an improvement in visual acuity of at least two lines on Snellen eye chart in all the patients.

## Discussion

The small population size is the main drawback of our study.

The mean postoperative IOP at 6 and 12 months was statistically significant lower than the corresponding preoperative values, and the mean percentage change from baseline was clinically significant. Despite the reduction in IOP values after phacoemulsification, the number of glaucoma medication did not change significantly, but it showed a slight increase. Our findings concur with the findings of previous studies that document improvement in glaucoma control after phacoemulsification [**16**,**17**]. However, the majority of studies enrolled patients with no glaucoma or with medically controlled glaucoma. Therefore, a direct comparison could not be made, the present study enrolling only patients with medically uncontrolled glaucoma.

Although reduction of IOP by a mean of 2.7 mmHg after cataract surgery was statistically significant, this small change is often not sufficient to treat the patient with uncontrolled POAG only by cataract surgery. Consequently, only 15.8% of the patients did not require further glaucoma surgery for lowering IOP to the desired target values.

Previous studies have shown that cataract lens removal improve glaucoma control by reducing IOP. Although a clear relationship between cause and effect has been recorded and checked, the mechanisms by which cataract surgery influences IOP are not fully understood. Possible mechanisms can include the following: reduced aqueous humor secretion, reduced resistance to aqueous humor efflux, altered biomechanical barrier or blood-aqueous humor barrier [**3**]. Other studies demonstrate IOP reduction after cataract surgery in patients without associated ocular pathology [**18**,**19**]. This may bring some information regarding pressure reduction mechanisms. If IOP decrease would be caused by the increase in trabecular outflow, we would expect a greater decrease of IOP in eyes without glaucoma compared to patients with POAG, because in the latter, the trabeculum is compromised. Therefore, if reduction in IOP would be associated with reduced resistance to flow through the trabecular meshwork, the decrease in IOP may be due to increased uveoscleral rather than trabecular outflow [**3**]. Other possible mechanisms are decreasing aqueous humor secretion due to traction on the ciliary body determined by the postoperator contracted capsule through the zonular fibers, or altered biomechanical barrier or blood - aqueous humor barrier [**20**,**21**].

The present study confirms that cataract surgery with intraocular lens implantation lowers IOP. The group with the highest preoperative IOP had the greatest decrease in postoperative IOP. This demonstrates that the decrease in IOP after phacoemulsification is proportional with the value of preoperative IOP and the eyes that required the greatest reduction have benefited most. These results demonstrate that phacoemulsification with foldable IOL implantation can be considered as an option for treatment in eyes with glaucoma and high IOP.

Our findings suggest that sutureless clear corneal phacoemulsification with foldable intraocular lens implantation can be considered as the first option in patients with fairly uncontrolled primary open angle glaucoma. Of the 6 patients who have not required further glaucoma surgery, 5 are part of the group with low pressure values ranged between 21 and 23 mmHg. In this group, 26% of the patients did not require further glaucoma surgery.

Cataract surgery alone is not a good option in terms of medically uncontrolled POAG management. In this study, phacoemulsification was followed by glaucoma surgery in 84.2% of cases. For patients with uncontrolled POAG and cataract, the surgical treatment for both conditions could be considered. This could be done as combined surgery (phacotrabeculectomy) or staged approach (phacoemulsification followed by glaucoma surgery or the other way around). Cataract surgery alone compared to combined procedures has the advantage of providing rapid visual rehabilitation with fewer complications.

 The present study was done on eyes with preoperative medically uncontrolled glaucoma, therefore the extrapolation of the results to eyes with preoperative medically controlled glaucoma cannot be considered.

## References

[1] Lau JTF, Lee V, Fan D (2002). Knowledge about cataract, glaucoma, and age related macular degeneration in the Hong Kong Chinese population. Br J Ophthalmol.

[2] Friedman DS, Jampel HD, Lubomski LH (2002). Surgical strategies for coexisting glaucoma and cataract: an evidence-based update. Ophthalmology..

[3] Mathalone N, Hyams M, Neiman S (2005). Long-term intraocular pressure control after clear corneal phacoemulsification in glaucoma patients.. J Cataract Refract Surg.

[4] Suzuki R, Tanaka K, Sagara T (1994). Reduction of intraocular pressure after phacoemulsification and aspiration with intraocular lens implantation.. Ophthalmologica.

[5] Suzuki R, Kuroki S, Fujiwara N (1997). Ten-year follow-up of intraocular pressure after phacoemulsification and aspiration with intraocular lens implantation performed by the same surgeon. Ophthalmologica.

[6] Pera¨salo R (1997). Phaco-emulsification of cataract in eyes with glaucoma. ActaOphthalmolScand.

[7] Lai JS, Tham CC, Chan JC (2006). The clinical outcomes of cataract extraction by phacoemulsification in eyes with primary angle-closure glaucoma (PACG) and co-existing cataract: a prospective case series.. J Glaucoma.

[8] Jacobi PC, Dietlein TS, Luke C (2002). Primary phacoemulsification andintraocular lens implantation for acute angle-closure glaucoma. Ophthalmology.

[9] Kubota T, Toguri I, Onizuka N (2003). Phacoemulsification and intraocular lensimplantation for angle closure glaucoma after the relief of papillary block. Ophthalmologica.

[10] Groessl EJ, Liu L, Sklar M (2012). Measuring the impact of cataract surgery on generic and vision-specific quality of life. Qual Life Res.

[11] Minna NG, Sample PA, Pascual JP (2012). Comparison of Visual Field Severity Classification Systems for Glaucoma. J Glaucoma.

[12] Kass MA, Heuer DK, Higginbotham EJ (2002). The Ocular Hypertension Treatment Study: A randomized trial determines that topical ocular hypotensive medication delays or prevents the onset of primary open-angle glaucoma. Arch Ophthalmol.

[13] Minna J (2011). Comparison of Visual Field Severity Classification Systems for Glaucoma. Glaucoma.

[14] Lichter PR, Musch DC, Gillespie BW (2001). Interim clinical outcomes in the Collaborative Initial Glaucoma Treatment Study comparing initial treatment randomized to medications or surgery. CIGTS Study Group.. Ophthalmology.

[15] Janz NK, Wren PA, Lichter PR (2001). The Collaborative Initial Glaucoma Treatment Study: interim quality of life findings after initial medical or surgical treatment of glaucoma. CIGTS Study Group. Ophthalmology.

[16] Hayashi K, Hayashi H, Nakao F (2001). Effect of cataract surgery on intraocular pressure control in glaucoma patients. J Cataract Refract Surg.

[17] Shingleton BJ, Gamell LS, O’Donoghue MW (1999). Long-term changes in intraocular pressure after clear corneal phacoemulsification: normal patients versus glaucoma suspect and glaucoma patients. J Cataract Refract Surg.

[18] Pohjalainen T, Vesti E, Uusitalo RJ (2001). Intraocularpressure after phacoemulsification and intraocular lens implantation in nonglaucomatous eyes with and without exfoliation.. J Cataract Refract Surg.

[19] Schwenn O, Dick HB, Krummenauer F (2001). Intraocularpressure after small incision cataract surgery: temporal sclerocorneal versus clear corneal incision. J Cataract Refract Surg.

[20] Law SK, Riddle J (2011). Management of cataracts in patients with glaucoma. International Ophthalmol Clinics.

[21] Poley BJ, Lindstrom RL, Samuelson TW (2008). Long term effects on phacoemulsification with intraocular lens implantation in normotensive and ocular hypertensive eyes. J Cataract Refractive Surg.

